# Expression of Cystatin SN significantly correlates with recurrence, metastasis, and survival duration in surgically resected non-small cell lung cancer patients

**DOI:** 10.1038/srep08230

**Published:** 2015-02-04

**Authors:** Xun Cao, Yong Li, Rong-Zhen Luo, Lin Zhang, Song-Liang Zhang, Jun Zeng, Yu-Jing Han, Zhe-Sheng Wen

**Affiliations:** 1 Sun Yat-Sen University Cancer Center, State Key Laboratory of Oncology in South China, Collaborative Innovation Center of Cancer Medicine, Guangzhou, China; 2 Department of Pathology, Sun Yat-Sen University Cancer Center, Guangzhou, China; 3 Department of Clinical Laboratory, Sun Yat-Sen University Cancer Center, Guangzhou, China; 4 Department of Preclinical Medicines, Sun Yat-Sen University Cancer Center, Guangzhou, China; 5 Department of Thoracic Oncology, Sun Yat-Sen University Cancer Center, Guangzhou, China

## Abstract

Cystatin SN has been considered to be involved in human cancer, but its clinical significance in non-small cell lung cancer (NSCLC) has not been elucidated. The aim of this study was to evaluate the clinical value of Cystatin SN expression in patients with surgically resected NSCLCs. A retrospective analysis of 174 patients with surgically resected NSCLCs from April 2002 to March 2005 was performed with immunohistochemistry and fluorescence *in situ* hybridization to analyze the protein expression and amplification of *Cystatin SN*. The associations between Cystatin SN expression and recurrence, metastasis, and survival were investigated. In recurrence and metastasis analysis, compared with low-Cystatin SN expression NSCLCs, high expression tumors were more likely to recur and metastasize (*P* < 0.001). Disease-free survival (DFS) and overall survival (OS) were significantly prolonged in the low-Cystatin SN expression subgroup compared with the high-Cystatin SN expression subgroup (DFS, *P* < 0.001; OS, *P* = 0.001). A multivariate analysis confirmed that high expression of Cystatin SN was associated with poor survival (DFS, *P* = 0.001; OS, *P* = 0.006) and was an independent prognostic indicator. The present study indicates that high expression of Cystatin SN is a significant prognostic indicator of a higher rate of recurrence, metastatic risk, and poor survival in patients with surgically resected NSCLCs.

Lung cancer continues to be the most common cause of cancer deaths worldwide^1^. Patients with non-small cell lung cancer (NSCLC), representing 75%–80% of total lung cancer cases, carry a 5-year survival rate of 10%–15% for all stages. Despite efforts to improve the survival of patients with NSCLC, satisfactory outcomes have not been achieved. The most common problems that patients encounter are tumor recurrence and metastasis, which may lead to mortality after surgical resection^2^,^3^. Studies conducted several years ago have suggested that adjuvant cisplatin-based chemotherapy could provide a survival benefit for patients with completely resected stage II-IIIA NSCLC^4^. However, adjuvant chemotherapy is also associated with serious adverse side effects. Thus, a better understanding of the biochemical signaling pathways involved in NSCLC might allow us to identify valuable prognostic factors, select high-risk patients, provide appropriate adjuvant therapies and improve the prognoses of patients.

The proteolytic activities of cysteine proteases are controlled by specific inhibitors that belong to the Cystatin (CST) superfamily, which includes the type 1 Cystatins (stefins), type 2 Cystatins and the kininogens^5^. It has been suggested that stefins and Cystatins play roles in several diseases, including cancer, and are associated with alterations in the proteolytic system^6^. For example, an immunohistochemical analysis of breast cancer samples suggested that the risk of cancer-related death was significantly higher in patients with Cystatin A-positive tumors than in those with Cystatin A-negative tumors^7^. Cystatin C has been correlated with tumor metastasis and invasion and is associated with a high risk of death in patients with colorectal cancer^8^,^9^.

Cystatin SN (CST1), a member of family 2 within the CST superfamily and encoded by the *CST1* gene, plays an important role in the processes of inflammation and tumorigenesis^10^. Choi et al. have shown that Cystatin SN is an important participant in the regulation of proteolytic activity and is highly involved in gastric tumorigenesis through T cell factor (TCF)-mediated proliferative signaling^11^. In addition, Cystatin SN has also been identified as a novel tumor biomarker for colorectal carcinoma^12^. However, to the best of our knowledge, the relationships between Cystatin SN expression and the recurrence/metastasis and/or prognoses of NSCLCs are of limited number and scope. Therefore, the primary objective of the current study was to examine and evaluate the expression of Cystatin SN in patients with NSCLC. Furthermore, the overarching goal of this research is to estimate the predictive impact of Cystatin SN expression on recurrence, metastasis, and survival in patients with surgically resected NSCLC.

## Results

### Patient characteristics

Table 1 summarizes the baseline characteristics of the study population. The median age of the patients was 60 years (range, 30 to 79 years). Of the 174 patients with NSCLC, 76 (43.7%) were diagnosed with squamous cell carcinoma (SCC), and 98 (56.3%) were diagnosed with adenocarcinoma. A total of 65 (37.4%), 58 (33.3%), and 51 (29.3%) patients were diagnosed with stage I, stage II, and stage III disease, respectively. A total of 120 patients (70%) received four to six cycles of tri-weekly cisplatin-based adjuvant chemotherapy.

### Immunohistochemical assessment of Cystatin SN expression

**Figure 1:**
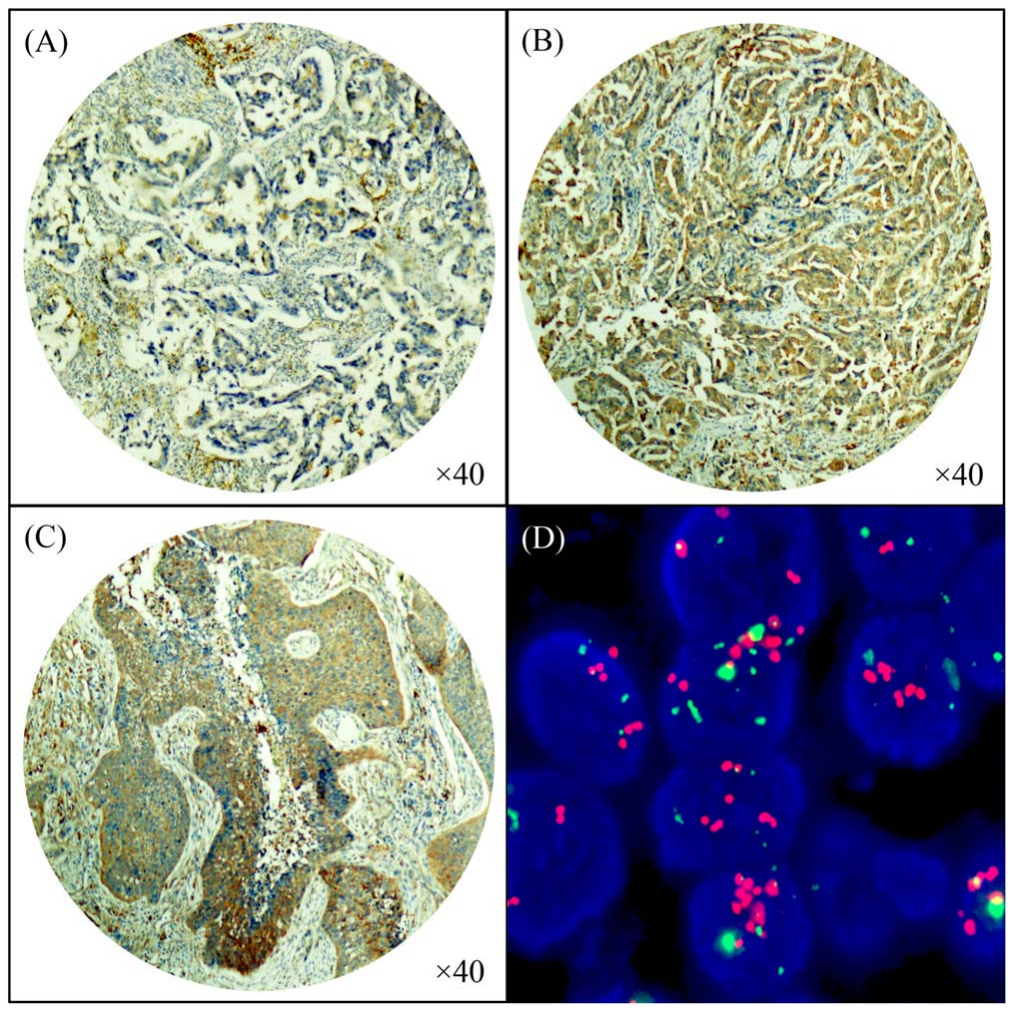
Immunohistochemical staining of Cystatin SN protein in non-small cell lung cancer. (A) Adenocarcinoma with low Cystatin SN expression; (B) adenocarcinoma with high Cystatin SN expression; (C) squamous cell carcinoma with high Cystatin SN expression; and (D) amplification of the Cystatin SN gene according to FISH in adenocarcinoma tissue.

Figure 1(A)–(C) shows that the expression of Cystatin SN was localized to the cytoplasm. Based on the criteria described, 89 (51.1%) of the 174 tumors exhibited high Cystatin SN expression, and 85 (48.9%) exhibited low Cystatin SN expression. Table 1 shows the comparison of the clinicopathological parameters according to the expression levels of Cystatin SN protein. The χ^2^ test showed that high levels of Cystatin SN expression were closely correlated with visceral pleural invasion (*P* = 0.039, more common in the presence than in the absence of visceral pleural invasion). Logistic regression further confirmed the correlation between high Cystatin SN expression and visceral pleural invasion (*P* = 0.041) (Table 2). No significant associations were observed between Cystatin SN expression and the other clinicopathological characteristics, including age, gender, tumor laterality, histology, tumor differentiation, pathological tumor status, pathological node status, pathological TNM status and adjuvant chemotherapy (χ^2^ test, *P* > 0.05).

### Cystatin SN expression and recurrence or distant metastasis

**Figure 2:**
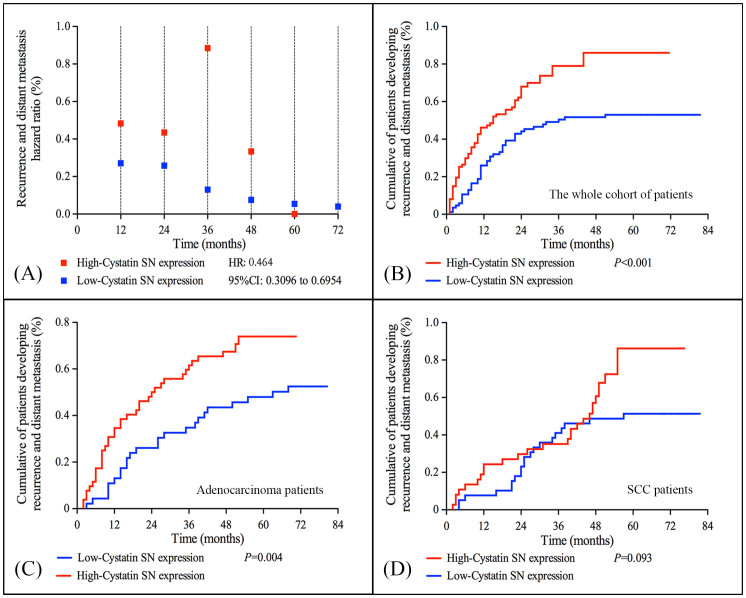
Hazard ratio analyses of recurrence and distant metastasis in patients with non-small cell lung cancer. (A) Hazard ratio analyses of annual recurrence and distant metastasis for patients with NSCLC according to the expression level of Cystatin SN protein; (B–D) Comparison of the cumulative percentage of patients who developed recurrence and distant metastasis between those with high and low Cystatin SN expression.

The annual recurrence or distant metastasis hazard ratio curve for patients with high Cystatin SN expression showed a peaked pattern with a major recurrence and distant metastasis surge that reached the maximum during the third year after primary treatment. Compared with patients with high Cystatin SN expression, the recurrence and distant metastasis curve for patients with low Cystatin SN expression was lower and flatter [Fig. 2(A)]. Additionally, for the whole cohort, a significant difference was observed in the cumulative percentages of patients who presented recurrence and distant metastasis between those with high Cystatin SN expression and those with low expression (*P* < 0.001) [Fig. 2(B)]. We also observed a significant different in cumulative percentage of patients developing recurrence and distant metastasis in adenocarcinoma patients who had high and low Cystatin SN expression (*P* = 0.004), whereas this was not significantly different between high and low Cystatin SN groups in patients with SCC (*P* = 0.093) [Fig. 2(C–D)].

### Cystatin SN expression and survival

**Figure 3:**
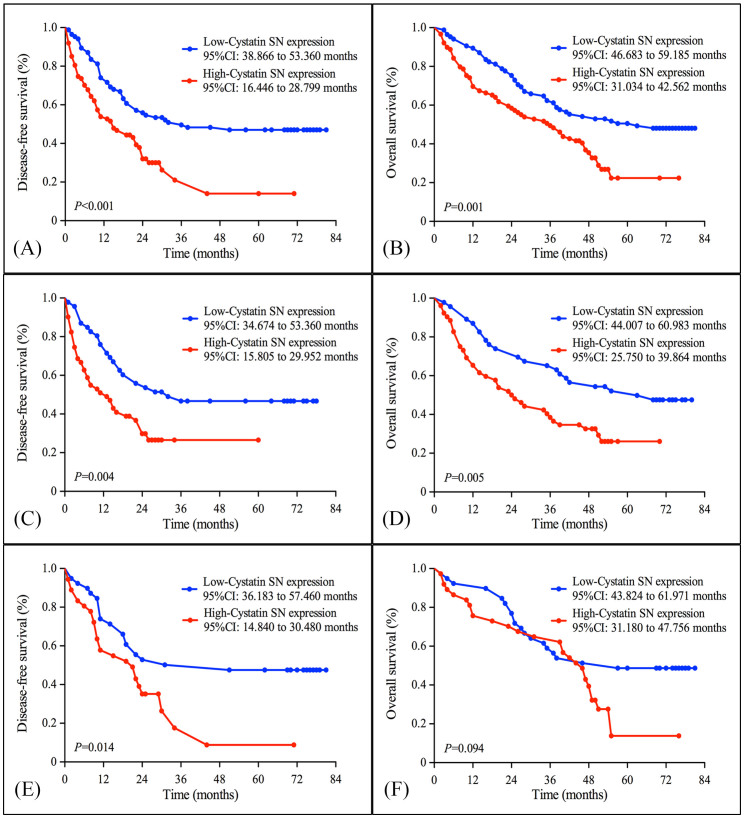
Kaplan-Meier estimates of the probability of survival in patients with non-small cell lung cancer. (A) Disease-free survival (DFS) and (B) Overall survival (OS) curves for the whole cohort of patients with NSCLC; (C) DFS and (D) OS curves for the adenocarcinoma patients; (E) DFS and (F) OS curves for the SCC patients according the Cystatin SN protein expression level.

All patients were included in the survival analysis. The median follow-up period was 41.5 months. A total of 67 patients were alive and 107 cancer-related deaths had occurred at the time of the last clinical follow-up. The five-year survival probability was 22% for the high Cystatin SN expression subgroup and 48% for the low Cystatin SN expression subgroup. According to the Kaplan-Meier analysis, the expression level of Cystatin SN was significantly associated with DFS and OS. For the full cohort, the median DFS was longer in patients with low Cystatin SN expression compared with those with high Cystatin SN expression (36 months *vs*. 15 months, *P* < 0.001) [Fig. 3(A)]. The median OS was 63 months for patients with low Cystatin SN expression and 36 months for patients with high Cystatin SN expression (*P* = 0.001) [Fig. 3(B)]. Furthermore, when we carried out the survival analyses in each histology subgroup, the benefit of low Cystatin SN expression was significant in patients with adenocarcinoma (DFS, 32 *vs.* 13 months, *P* = 0.004; OS, 63 *vs.* 24 months, *P* = 0.005) [Fig. 3(C–D)], whereas OS duration was similar between high and low-Cystatin SN expression subsets in SCC patients (57 *vs.* 46 months, *P* = 0.094) [Fig. 3(E–F)]. To determine whether Cystatin SN protein expression could serve as an independent prognostic parameter, we examined DFS and OS using univariate and multivariate Cox proportional hazards models. The results revealed that Cystatin SN expression (DFS, *P* = 0.001; OS, *P* = 0.006), pTNM stage (DFS, *P* < 0.001; OS, *P* < 0.001) and adjuvant chemotherapy (DFS, *P* < 0.001; OS, *P* < 0.001) were independent, significant predictors of DFS and OS. The details of these results are presented in Table 3 and Table 4.

### Amplification of *Cystatin SN* in NSCLCs

A total of 74 (91.4%) NSCLC samples showed both high Cystatin SN protein expression and the amplification of *Cystatin SN* [Fig. 1(D)]. The χ^2^ test results demonstrated a significant association between high Cystatin SN expression and the amplification of *Cystatin SN* (*P* < 0.001, Table 5). However, high Cystatin SN protein expression was also detected in 16.1% (*n* = 15) of the NSCLC samples in the absence of Cystatin SN amplification.

## Discussion

Although growing evidence has supported the role of Cystatin SN in tumor invasion and metastasis, investigators have placed little emphasis on the clinical and prognostic significance of Cystatin SN. In the present study, we have demonstrated for the first time that the high Cystatin SN expression subtype NSCLC is more frequent when pleural invasion occurs, compared with the low Cystatin SN expression subtype. In addition, comparison between patients with low Cystatin SN expression and those with high Cystatin SN expression indicated that the latter were more likely to experience recurrence/metastasis, which was associated with a greater hazard ratio peak. Most importantly, Cystatin SN expression is associated with increased survival and is an independent prognostic parameter for survival in patients with surgically resected NSCLCs. These findings indicate that Cystatin SN expression may promote the malignant properties of tumor cells, leading to a poor prognosis in patients with NSCLC.

Cystatin SN is a 121-amino acid (a.a.) protein and an active cysteine protease inhibitor of the CST superfamily. A number of studies have suggested that Cystatin SN is a novel biomarker for human cancer. In a study by Yoneda et al., Cystatin SN was identified as a tumor marker for colorectal cancer^12^. They observed that *Cystatin SN* mRNA and protein were specifically up-regulated in colorectal cancer cell lines (3-fold) and tissues, respectively. Furthermore, Cystatin SN protein was elevated in the serum and urine of patients with colorectal cancer compared with healthy controls (1.70 ng/ml *vs.* 1.12 ng/ml). Choi and colleagues reported that the mRNA expression of *Cystatin SN* was markedly increased in gastric cancer tissues compared with normal paracancerous tissues; clinicopathological analyses revealed a significant association between high Cystatin SN expression and the pTNM stage^11^. In addition, cell proliferation increased 1.3-fold in Cystatin SN-overexpressing AGS cells compared with GFP-vector control cells and SNU638 cells^11^. After AGS cells were transfected with *Cystatin SN*-small interfering RNA (siRNA) (to suppress the level of *Cystatin SN* transcript) or control siRNA, the number of cells transfected with *Cystatin SN*-siRNA were fewer in number than those of the control siRNA-transfected cells. These findings support the inference that of Cystatin SN expression might provide important information regarding the prognosis of patients with NSCLC.

The association of Cystatin SN expression with malignant behaviors, including proliferation and metastasis, indicates its biological involvement in human cancer. However, the molecular mechanisms underlying the role of Cystatin SN in these processes remain unknown. We believe that the changes in the stability and equilibrium of cysteine protease/cysteine protease inhibitor complexes could contribute to the prognosis of cancer patients. For example, the overexpression of Cystatins, combined with normal expression levels of cysteine protease inhibitor in tumor cells, may augment the metastatic potential of a cancer via protection of the tumor cells against tumor necrosis factor (TNF)-mediated apoptosis^13^. Foghsgaard et al. showed that TNF-induced apoptosis of tumor cells is mediated by a caspase-independent pathway in which cysteine protease inhibitors function as major execution proteases^14^. Notably, ectopic overexpression of Cystatin A rescued tumor cells from TNF-induced apoptosis^14^. Overexpression of Cystatins may also enhance the metastatic potential of tumor cells by rescuing them from NK cell-mediated apoptosis^13^. Aside from TNF-related and NK cell-mediated apoptosis signaling pathways, several studies have demonstrated that Cystatins appear to block downstream apoptotic pathways, for example, by disrupting mitochondrial integrity and the release of cytochrome *c*^13^,^15^,^16^. All of these findings have supported the idea that Cystatin-mediated inhibition of cysteine proteases leads to a reduction in apoptotic cell death in cancer. Furthermore, with regard to cysteine protease inhibitors, it has been proposed that besides protease inactivation, additional tumorigenic functions contribute to a worse prognosis^17^. In the study by Kuopio et al. in breast cancer, high expression of Cystatin A was found to positively correlate with tumor size, increased mitotic activity, and negative staining for the anti-apoptotic protein Bcl-2^7^. The above studies and our results all offer evidence that *Cystatins* may function as proliferation-, apoptosis-, and metastasis-related oncogenes in tumorigenesis. Thus, the expression level of Cystatin SN protein might provide information regarding the likelihood of recurrence and metastasis in, as well as the survival of, patients with NSCLC and identify patients who might actually benefit from multimodal therapies.

With regard to the mechanism underlying up-regulated expression of Cystatin SN protein in NSCLCs, it is known that the high expression of an oncogene is often caused by DNA amplification^18^. To determine whether the high expression of Cystatin SN protein in NSCLCs is caused by gene amplification, the amplification status of *Cystatin SN* was examined by FISH. Our results suggested that DNA amplification might be one of several mechanisms responsible for a high expression level of Cystatin SN protein because high Cystatin SN protein expression was also observed in a portion of tumor tissues without *Cystatin SN* amplification. Thus, we propose that the regulation of Cystatin SN protein expression is quite complicated. The expression of Cystatin SN may be regulated not only by gene amplification but also by other molecular mechanisms, including transcriptional regulation.

We also acknowledged the limitations of the present retrospective analysis. First, the expression level of Cystatin SN could not be determined using reverse transcription-PCR (rt-PCR) due to the lack of fresh frozen tissues. Thus, a further investigation will include rt-PCR expression analysis of Cystatin SN and will be performed to confirm the present findings. Second, we did not examine the protein expression levels of the upstream and/or downstream effectors of Cystatin SN, which could provide further evidence of the functional status of Cystatin SN. In addition, our study is a retrospective study that relied exclusively on a single-institutional database. Additional investigations of the biological significance of Cystatin SN expression in cell lines and animal models must be performed.

Collectively, this is the first study to show that patients with completely resected NSCLCs and high Cystatin SN protein expression in their tumors had a higher risk of recurrence/metastasis and poorer survival compared with patients with low Cystatin SN expression. Our findings demonstrate that the levels of Cystatin SN protein expression in NSCLCs after radical surgery can serve as an independent predictor of patient outcomes.

## Methods

### Patients

From April 2002 until March 2005, a total of 174 patients at the Sun Yat-Sen University Cancer Center who underwent complete surgical resection (complete resection of lung cancer and mediastinal lymph node dissection with microscopic examination of the tumor-free margins) for NSCLC were eligible for our study. The pretreatment evaluation included a complete history and physical examination, complete blood cell count, serum biochemistry (including renal function test), chest X-ray, computed tomography (CT) scans of the chest and abdomen, and bronchoscopy. Whole-body bone scans and magnetic resonance imaging (MRI) scans of the brain were used to exclude patients with possible metastases. Patients with previous malignancies, a second primary tumor, or a suspected distant metastasis, as well as those who received preoperative treatment (chemotherapy and/or radiotherapy), were excluded. Adjuvant chemotherapy (cisplatin-based combinations) was given to patients with stage IB to III disease if the patients were able to tolerate treatment after curative-intent surgery, unless the patients refused adjuvant chemotherapy. Patients with stage III disease and pathological evidence of N2 disease received postoperative mediastinal radiotherapy. When one or more recurrence/metastasis was diagnosed after completion of the primary treatment, chemotherapy was administered if the patient exhibited a performance status that indicated the ability to tolerate the treatments. Moreover, some patients received palliative radiotherapy in addition to chemotherapy. The histological diagnosis and tumor differentiation grades were defined according to the World Health Organization/International Association for the Study of Lung Cancer (WHO/IASLC) criteria^19^. The stage was recorded based on the IASLC staging system^20^. This study was approved by the medical ethics committee of Sun Yat-Sen University Cancer Center. Informed consent was obtained from all patients prior to specimen collection. All experiments were performed in accordance with approved guidelines and regulations.

After the completion of primary treatment, patients were followed up every 4–6 months during the first 3 years and every 12 months thereafter. The survival status of the patients was verified again with the available methods in May 2010, including verification of the clinical attendance records and direct telecommunications with the patients or their families.

### Construction of the tissue microarray

The tissue microarray (TMA) was constructed according to the methods described previously^21^. The tissues (174 NSCLC tissues and 20 normal lung tissues) from the tumor bank were collected, fixed in ethanol and embedded in paraffin. Hematoxylin and eosin-stained sections from a single random block from each patient were reviewed by a senior pathologist (Rong-Zhen Luo) to define representative tumor regions. Two targeted core samples of each specimen were obtained using a tissue array instrument (MiniCore; Alphelys, Plaisir, France). Briefly, 10-mm tissue cylinders were punched and arrayed on a recipient paraffin block. Sections (5 μm) of the tissue array (recipient) block were cut and placed on glass slides.

### Immunohistochemical staining for Cystatin SN

A standard protocol for immunostaining tissue microarray sections was used. In brief, tissue microarray sections were rehydrated with graded alcohol solutions. Endogenous peroxidase activity was blocked with 0.3% hydrogen peroxide for 15 minutes. For epitope retrieval, the tissue microassay slides were exposed to 10 mM citrate buffer (pH 6.0) and heated for 5 minutes. The tissue microarray slides were incubated with anti-Cystatin SN antibody at a dilution of 1:800 (NBP1-55995, Novus, Littleton, USA) for 12 hours at 4°C in a moist chamber. Subsequently, a biotinylated secondary antibody was applied for 30 minutes at 37°C. Then, the sections were incubated with streptavidin-horseradish peroxidase complex and developed with 3-diaminobenzidine tetrahydrochloride (DAB). Mayer's hematoxylin was applied as the counterstain. Cystatin SN-immunopositive sections of colon carcinoma were used as the positive control. As a negative control, the primary antibody was replaced with normal rabbit serum.

### Evaluation of immunohistochemical staining

The cytoplasmic Cystatin SN staining was graded by light microscopy to generate an immunoreactivity score (IRS)^22^,^23^. The IRS of Cystatin SN expression was calculated by multiplying the intensity by the extent score. The staining intensity was scored as 0 (negative), 1 (weak, light yellow), 2 (intermediate, yellow-brown) and 3 (strong, brown). The percentage of positively stained cells was evaluated as 0 (0%), 1 (1%–10%), 2 (11%–50%), 3 (51%–70%) and 4 (71%–100%). The IRS was classified as - (0, negative), 1+ (range from 1 to 4, weak), 2+ (range from 5–8, intermediate) and 3+ (range from 9–12, strong). For Cystatin SN expression, the IRSs of the normal lung tissues were 2+ to 3+. Thus, we defined an IRS of 0 to 1+ as low Cystatin SN expression and an IRS of 2+ to 3+ as high Cystatin SN expression.

Two investigators who were blinded to the clinicopathological data independently evaluated Cystatin SN staining by light microscopy. In the present study, at least 300 epithelial cells were counted for each normal or tumor case. To ensure the consistency of the scores, discordant cases were reviewed.

### Fluorescence *in-situ* hybridization (FISH)

Two-color fluorescence *in-situ* hybridization was performed with a Spectrum Orange-labeled BAC clone at chromosome 20p11 that contained the *Cystatin* gene and a Spectrum Green-labeled reference centromeric probe on chromosome 20 (TelVysion 20p Spectrum Green, 05J03-020, 20pTEL18, Vysis, Abbott). The deparaffinized tissue sections were treated with proteinase K (400 μg/ml) at 37°C for 45 minutes, followed by denaturation in 70% formamide and 2 × SSC at 75°C for 8 minutes. A total of 50 ng of each probe was mixed in a 10-μl hybridization mixture (containing 55% formamide, 2 × SSC, and 2 g human Cot1 DNA). This mixture was denatured at 75°C for 6 minutes and then hybridized to the denatured TMA section at 37°C for 24 hours. After washing, the section was counterstained with 1 μg/ml DAPI in an anti-fade solution and examined with a Zeiss Axiophot microscope equipped with a triple-band pass filter. FISH signals from 300 cells in each sample were counted. The criteria for *Cystatin SN* gene amplification were defined as the presence of either 6 (or more) gene signals or more than 3 times as many gene signals than centromere signals on chromosome 20.

### Statistical analysis

The correlations between Cystatin SN expression and the clinicopathological parameters of patients with NSCLC were assessed with the χ^2^ test. Logistic regression was performed to further identify the possible role of Cystatin SN expression with regard to the clinicopathological parameters. The disease-free survival (DFS) and the overall survival (OS) were calculated in months from the date of surgery to the date of regional recurrence or distant metastasis (for DFS) and death or final clinical follow-up (for OS). The DFS and OS were analyzed using the Kaplan-Meier method and compared using the log-rank test. A multivariate Cox proportional hazards regression analysis was performed for all variables found to be significant in a univariate analysis. Two-sided *P* < 0.05 was considered statistically significant. The statistical analyses were performed with the SPSS 13.0 software package (SPSS, Chicago, IL) and GraphPad Prism (Version 5, GraphPad Software, Inc.).

## Author Contributions

X.C., Y.L. and R.Z.L. performed the data acquisition and the statistical analysis, drafted the manuscript and participated in the sequence alignment. L.Z. participated both in the design of the study and in the sequence alignment. S.L.Z., J.Z. and Y.J.H. participated in the sequence alignment and performed the data acquisition. Z.S.W. conceived the study, participated in its design and coordination and helped draft the manuscript. All authors read and approved the final manuscript.

## Figures and Tables

**Table 1:** Characteristics of the Patients

Characteristic	All Patients (*n* = 174)		Patient with Cystatin SN-low tumors (*n* = 85, percent)		Patients with Cystatin SN-high tumors (*n* = 89, percent)		*P* value*
Age (years)							0.285
Median, Range	60	30–79	61	36–77	58	30–79	
Gender							0.868
Male	134		65	(48.5)	69	(51.5)	
Female	40		20	(50)	20	(50)	
Tumor Laterality							0.381
Left	63		28	(44.4)	35	(55.6)	
Right	111		57	(51.4)	54	(48.6)	
Histology							0.567
Adenocarcinoma	98		46	(46.9)	52	(53.1)	
Squamous Cell carcinoma	76		39	(51.3)	37	(48.7)	
Visceral Pleural Invasion							0.039
Absent	47		29	(61.7)	18	(38.8)	
Present	127		56	(44.1)	71	(55.9)	
Tumor Differentiation							0.977
Grade 1	13		6	(46.2)	7	(53.8)	
Grade 2	77		38	(49.4)	39	(50.6)	
Grade 3	84		41	(48.8)	43	(51.2)	
Pathological Tumor Status							0.391
T1	26		16	(61.5)	10	(38.5)	
T2	126		58	(46)	68	(54)	
T3	16		9	(56.2)	7	(43.8)	
T4	6		2	(33.3)	4	(66.7)	
Pathological Node Status							0.532
N0	92		47	(51.1)	45	(48.9)	
N1/2 (N_metastasis_)	82		38	(46.3)	44	(53.7)	
Pathological TNM Status							0.205
Stage I	65		36	(55.4)	29	(44.6)	
Stage II	58		23	(39.7)	35	(60.3)	
Stage III	51		26	(51)	25	(49)	
Adjuvant Chemotherapy							0.435
Yes	120		61	(50.8)	59	(49.2)	
No	54		24	(44.4)	30	(55.6)	

Abbreviation: T, tumor; N, node; TNM, tumor-node-metastasis.

*χ^2^ test.

**Table 2:** Logistic regression analyses with the expression level of Cystatin SN

Variable	*b*	HR	95%CI	*P* value*
Visceral Pleural Invasion				0.041
Absent *vs.* Present	0.714	2.043	1.030–4.050	

Abbreviation: HR, hazard ratio; 95%CI, 95% confidence interval.

*Logistic regression, forward stepwise selection method.

**Table 3:** Univariate Cox regression Analysis for Disease-free Survival and Overall Survival in Patients with Non-Small Cell Lung Cancer

	Disease-free survival		Overall survival	
	HR (95%CI)	*P* value	HR (95%CI)	*P* value
Age (years)	1.012 (0.993–1.030)	0.217	1.013 (0.994–1.032)	0.193
Gender		0.860		0.925
Male	1.000		1.000	
Female	0.961 (0.618–1.495)		0.925 (0.595–1.439)	
Tumor Laterality		0.995		0.824
Left	1.000		1.000	
Right	1.001 (0.675–1.485)		1.046 (0.705–1.551)	
Histology		0.521		0.481
Adenocarcinoma	1.000		1.000	
SCC	1.134 (0.773–1.663)		1.148 (0.783–1.684)	
Pleural Invasion		0.104		0.110
Absent	1.000		1.000	
Present	1.458 (0.926–2.297)		1.448 (0.919–2.282)	
Tumor Differentiation		0.430		0.328
Grade 1	1.000		1.000	
Grade 2	0.884 (0.397–1.796)		0.791 (0.372–1.683)	
Grade 3	1.098 (0.523–2.305)		1.069 (0.509–2.243)	
pTNM Status		<0.001		<0.001
Stage I	1.000		1.000	
Stage II	3.145 (1.890–5.232)		2.982 (1.795–4.955)	
Stage III	3.647 (2.172–6.123)		3.387 (2.020–5.679)	
Adjuvant Chemotherapy		0.006		0.017
Yes	1.000		1.000	
No	1.762 (1.178–2.634)		1.629 (1.092–2.430)	
Cystatin SN Expression		<0.001		0.001
Low	1.000		1.000	
High	2.099 (1.412–3.121)		1.906 (1.284–2.830)	

Abbreviation: HR, hazard ratio; 95%CI, 95% confidence interval; SCC, squamous cell carcinoma; pTNM status, pathological Tumor-Node-Metastasis.

**Table 4:** Multivariate Cox regression analysis for disease-free survival and overall survival in patients with non-small cell lung cancer

	Disease-free survival		Overall survival	
Factors	HR (95%CI)	*P* value*	HR (95%CI)	*P* value*
pTNM Status		<0.001		<0.001
Stage I	1.000		1.000	
Stage II	3.058 (1.821–5.134)		2.854 (1.703–4.782)	
Stage III	3.949 (2.325–6.710)		3.483 (2.062–5.884)	
Adjuvant Chemotherapy		<0.001		<0.001
Yes	1.000		1.000	
No	1.283 (0.763–2.158)		1.222 (0.729–2.050)	
Cystatin SN Expression		0.001		0.006
Low	1.000		1.000	
High	2.473 (1.457–4.074)		2.048 (1.231–3.406)	

Abbreviation: HR, hazard ratio; 95%CI, 95% confidence interval; SCC, squamous cell carcinoma; pTNM status, pathological Tumor-Node-Metastasis.

*Cox proportional hazards model.

**Table 5:** Association between Cystatin SN expression and amplification in patients with non-small cell lung cancer

			Cystatin SN expression		
			Low	High	
		Case	(*n* = 85, percent)	(*n* = 89, percent)	*P* value*
Cystatin SN	No	93	78 (83.9)	15 (16.1)	<0.001
Amplification	Yes	81	7 (8.6%)	74 (91.4)	

*χ^2^ test.
